# Whole-genome resequencing of three *Coilia nasus* population reveals genetic variations in genes related to immune, vision, migration, and osmoregulation

**DOI:** 10.1186/s12864-021-08182-0

**Published:** 2021-12-06

**Authors:** Jun Gao, Gangchun Xu, Pao Xu

**Affiliations:** 1 Wuxi Fisheries College, Nanjing Agricultural University, Wuxi, 214081 Jiangsu China; 2 Key Laboratory of Freshwater Fisheries and Germplasm Resources Utilization, Ministry of Agriculture, Freshwater Fisheries Research Center, Chinese Academy of Fishery Sciences, Wuxi, 214081 Jiangsu China

**Keywords:** Genome resequencing, *Coilia nasus* populations, Selective sweeping, Genetic variation, Migration

## Abstract

**Background:**

*Coilia nasus* is an important anadromous fish, widely distributed in China, Japan, and Korea. Based on morphological and ecological researches of *C. nasus*, two ecotypes were identified. One is the anadromous population (AP). The sexually mature fish run thousands of kilometers from marine to river for spawning. Another one is the resident population which cannot migrate. Based on their different habitats, they were classified into landlocked population (LP) and sea population (SP) which were resident in the freshwater lake and marine during the entire lifetime, respectively. However, they have never been systematically studied. Moreover, *C. nasus* is declining sharply due to overfishing and pollution recently. Therefore, further understandings of *C. nasus* populations are needed for germplasm protection.

**Results:**

Whole-genome resequencing of AP, LP, and SP were performed to enrich the understanding of different populations of *C. nasus*. At the genome level, 3,176,204, 3,307,069, and 3,207,906 single nucleotide polymorphisms (SNPs) and 1,892,068, 2,002,912, and 1,922,168 insertion/deletion polymorphisms (InDels) were generated in AP, LP, and SP, respectively. Selective sweeping analysis showed that 1022 genes were selected in AP vs LP; 983 genes were selected in LP vs SP; 116 genes were selected in AP vs SP. Among them, selected genes related to immune, vision, migration, and osmoregulation were identified. Furthermore, their expression profiles were detected by quantitative real-time PCR. Expression levels of selected genes related to immune, and vision in LP were significantly lower than AP and SP. Selected genes related to migration in AP were expressed significantly more highly than LP. Expression levels of selected genes related to osmoregulation were also detected. The expression of *NKAα* and *NKCC1* in LP were significantly lower than SP, while expression of *NCC*, *SLC4A4*, *NHE3*, *and V-ATPase* in LP was significantly higher than SP.

**Conclusions:**

Combined to life history of *C. nasus* populations, our results revealed that the molecular mechanisms of their differences of immune, vision, migration, and osmoregulation. Our findings will provide a further understanding of different populations of *C. nasus* and will be beneficial for wild *C. nasus* protection.

**Supplementary Information:**

The online version contains supplementary material available at 10.1186/s12864-021-08182-0.

## Background

The Chinese tapertail anchovy, *Coilia nasus*, is a commercially valuable fish, widely distributed in China, Japan, and Korea [1]. Based on morphological and ecological researches research of *C. nasus*, two ecotypes were identified [2, 3]. One is the anadromous population (also called river anchovy), with wide distribution in sea areas nearby China, Japan, and Korea. Before sexually mature, the anadromous population grows in coastal waters near the estuary. The sexually mature fish run thousands of kilometers from marine to river, such as the Yangtze River, to spawn from February [4]. During spawning migration, they generally do not feed, but a small part of *C. nasus* feed [5]. After reproduction, adults return to the marine. The eggs float down and hatch in the river and migrate the marine until they grow up to juveniles. The eggs float down and hatch in the river, and then the juveniles migrate the marine [4]. Another one is the resident population. They do not migrate. Another one is the resident population which cannot migrate. Based on their different habitats, one is lake anchovy, resident in the freshwater lake during the entire lifetime. Another one is sea anchovy, resident in marine during the entire lifetime.

The mechanisms of fish migration have not yet been elucidated. Many explanations of fish migration are only hypotheses that need to be verified. As an important behavioral feature, migration may be formed during evolution among many fish species or different geographic populations. This precise directional behavior of fish may be related to the sun, moon, aurora, geomagnetic field, water current, water temperature, or other environmental factors. It has been proved that European eel (*Anguilla anguilla*) use their magnetic compass to memorize the magnetic direction of tidal flows, which could help them to maintain their position in an estuary and to migrate upstream [6]. Migration may be affected by a single factor, or it may be affected by the combination of several factors. However, the specific factors affecting migration orientation are still unable to be determined. During migration, fishes own complex and sensitive sensory organs (such as vision, taste, lateral line system, etc.) and central nervous system plays essential role in receiving physical and chemical orientation information from the outside world, which makes the location and migration successful. Wisby and Hasler (1954) and Nordeng (1971, 1977) have proposed two olfactory hypotheses for imprinting and homing in silver salmon (*Oncorhynchus kisutch*), Arctic char (*Salvelinus alpinus*), and Atlantic salmon (*Salmo salar*) [7–9]. It has been concluded that functional olfactory ability is critical to accurate spawning migration in salmonids and American eels (*Anguilla rostrata*) [10–12]. It has been reported that olfaction may be involved in the spawning migration of *C. nasus* [13, 14]. Furthermore, recent studies showed that nerve and signal conduction in brain might participate in the regulation of spawning migration in *C. nasus* through transcriptomic analysis [15, 16].

Besides migration, other differences in biological characteristics among different *Coilia nasus* populations still need to explore. Thus, in the present study, we performed whole-genome resequencing of three *C. nasus* populations, anadromous population (AP), landlocked population (LP), and sea population (SP). Lots of single nucleotide polymorphisms (SNPs) and insertions and deletions (InDels) were generated. Then, selective sweeping analysis was used to identify selected genes. These genes were detected via quantitative real-time PCR (qRT-PCR). These identified genes will provide valuable resources for genetic research on *C. nasus*.

## Results

### Genome resequencing and mapped on reference genome

After filtering adaptors, ambiguous “N” nucleotides, and low-quality sequences, 276.78 G clean reads were generated, and the average sequencing depth was 10 ×. 296.61, 327.20, and 300.66 million clean reads were generated in AP, LP, and SP, respectively. The genomic GC contents of AP, LP, and SP were 43.907, 44.031, and 44.075%, respectively. The mapping rates between samples and reference genome were over 95% (Additional file 1: Table S1).

### Identification of SNPs and InDels of AP, LP, and SP

**Fig. 1:**
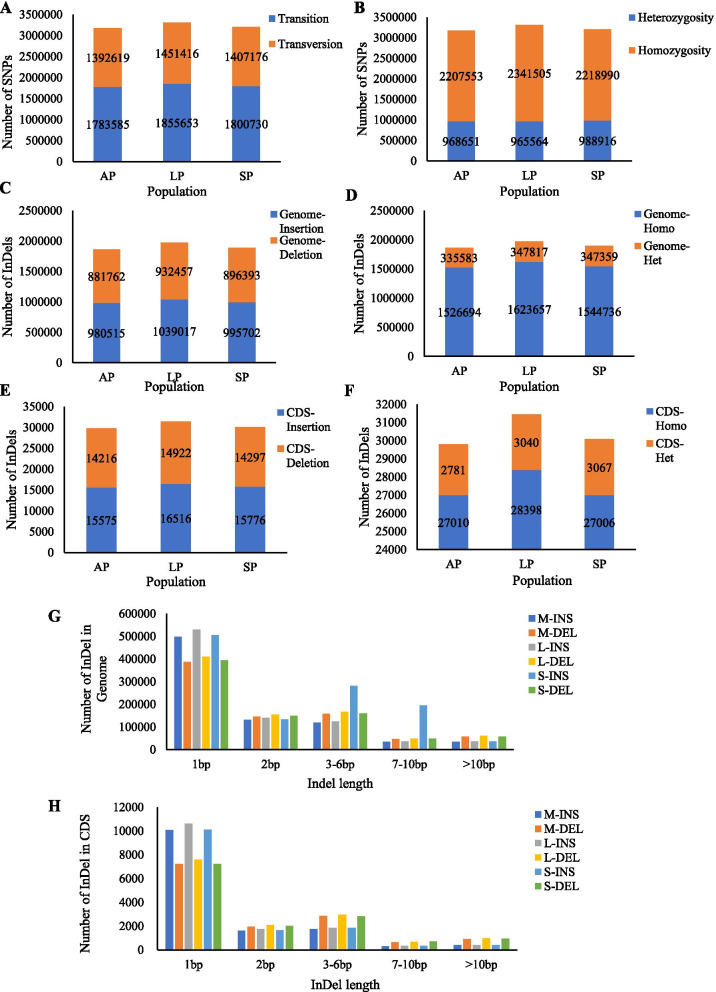
Statistics of SNPs and InDels. **A** SNP types. **B** Numbers of homologous and heterozygous SNPs. **C** Numbers of insertion and deletion on genome. **D** Numbers of homologous and heterozygous InDels on genome. **E** Numbers of insertion and deletion on coding sequences (CDS). **F** Numbers of homologous and heterozygous InDels on CDS. **G** Length distribution of InDels on genome. **H** Length distribution of InDels on CDS. Homo, Homozygosity; Het, Heterozygosity

Genome-wide SNPs of AP, LP, and SP were identified compared with reference genome. 3,176,204, 3,307,069, and 3,207,906 SNPs sites were identified in AP, LP, and SP, respectively. The mutation of SNP type is divided into two types: transition and transversion. The mutation between the same type of base is called transition (Ti). The mutation between the different types of bases is called transversion (Tv). In AP, 1783585 transitions and 1,392,619 transversions were found, and Ti/ Tv was 1.28 (Fig. 1A; Additional file 2: Table S2). In LP, 1855653 transitions and 1,451,416 transversions were found, and Ti/ Tv was 1.27 (Fig. 1A; Additional file 2: Table S2). In AP, 1800730 transitions and 1,407,176 transversions were found, and Ti/ Tv was 1.27 (Fig. 1A; Additional file 2: Table S2). For diploid or polyploid species, if a certain SNP site on a homologous chromosome is the same base, the SNP site is called a homozygous SNP site; if a SNP site on a homologous chromosome Contains different types of bases, the SNP site is called a heterozygous SNP site. In AP, 968651 heterozygous SNP sites and 2,207,553 homozygous SNP sites were found, and Het-radio was 30.49% (Fig. 1B; Additional file 2: Table S2). In LP, 965564 heterozygous SNP sites and 2,341,505 homozygous SNP sites were found, and Het-radio was 29.19% (Fig. 1B; Additional file 2: Table S2). In SP, 988916 heterozygous SNP sites and 2,218,990 homozygous SNP sites were found, and Het-radio was 30.82% (Fig. 1B; Additional file 2: Table S2).

Genome-wide InDels of AP, LP, and SP were identified compared with reference genome. In AP, total 1,862,277 InDels were found in genome, including 980,515 insertions and 881,762 deletions (Fig. 1C; Additional file 3: Table S3); 1,526,694 homozygosity and 335,583 heterozygosity (Fig. 1D; Additional file 3: Table S3). In LP, total 1,971,474 InDels were found in genome, including 1,039,017 insertions and 932,457 deletions (Fig. 1C; Additional file 3: Table S3); 1,623,657 homozygosity and 347,817 heterozygosity (Fig. 1D; Additional file 3: Table S3). In SP, total 1,892,095 InDels were found in genome, including 995,702 insertions and 896,393 deletions (Fig. 1C; Additional file 3: Table S3); 1,544,736 homozygosity and 347,359 heterozygosity (Fig. 1D; Additional file 3: Table S3). Furthermore, in AP, 29791 InDels were found in coding sequences (CDS), including 15,575 insertions and 14,216 deletions (Fig. 1E; Additional file 3: Table S3); 27,010 homozygosity and 2781 heterozygosity (Fig. 1F; Additional file 3: Table S3). In LP, 31438 InDels were found in CDS, including 16,516 insertions and 14,922 deletions (Fig. 1E; Additional file 3: Table S3); 28,398 homozygosity and 3040 heterozygosity (Fig. 1F; Additional file 3: Table S3). In SP, 30073 InDels were found in CDS, including 15,776 insertions and 14,297 deletions (Fig. 1E; Additional file 3: Table S3); 27,006 homozygosity and 3067 heterozygosity (Fig. 1F; Additional file 3: Table S3). Moreover, mononucleotides insertion/deletion on genome (Fig. 1G; Additional file 4: Table S4) and CDS (Fig. 1H; Additional file 5: Table S5) were the most in AP, LP, and SP.

### Annotation of SNPs and InDels of AP, LP, and SP

According to the location of the mutation site on the reference genome and the gene location information on the reference genome, the region where the mutation site occurs in the genome (intergenic region, gene region or CDS region, etc.), and the impact of the mutation (synonymous, non-synonymous mutations, etc.). Most SNPs were located intergenic in AP, and most SNPs located in CDS were synonymous coding (Table 1). SNPs of LP and SP displayed similar trends to AP (Table 1). Most InDels were located intergenic in AP, and most of InDels located in CDS were synonymous coding (Table 2). InDels of LP and SP displayed similar trends to AP (Table 2). Moreover, chromosomes distribution of SNPs and InDels in AP, LP, and SP were shown in Additional files 6, 7, 8: Fig. S1-S3.

**Table 1:** Annotation of SNPs located on genome

Region	Type	AP	LP	SP
–	INTERGENIC	1,360,676	1,416,909	1,375,094
–	INTRAGENIC	65,586	67,712	65,674
–	INTRON	1,097,057	1,142,780	1,107,386
–	UPSTREAM	234,197	243,993	237,352
–	DOWNSTREAM	234,933	244,234	236,945
–	SPLICE_SITE_ACCEPTOR	281	302	282
–	SPLICE_SITE_DONOR	385	384	374
–	SPLICE_SITE_REGION	10,238	10,490	10,382
CDS	START_LOST	131	140	131
CDS	SYNONYMOUS_CODING	85,675	89,775	86,776
CDS	NON_SYNONYMOUS_CODING	48,144	50,097	48,283
CDS	SYNONYMOUS_STOP	55	53	49
CDS	STOP_GAINED	562	607	566
CDS	STOP_LOST	90	97	95

**Table 2:** Annotation of InDels located on genome

Region	Type	AP	LP	SP
–	INTERGENIC	743,775	790,990	758,116
–	INTRAGENIC	27,724	29,082	27,936
–	INTRON	571,084	603,941	580,111
–	UPSTREAM	115,552	121,665	117,912
–	DOWNSTREAM	118,897	124,840	120,153
–	SPLICE_SITE_ACCEPTOR	492	518	504
–	SPLICE_SITE_DONOR	511	535	545
–	SPLICE_SITE_REGION	3972	4163	4026
CDS	START_LOST	49	50	45
CDS	FRAME_SHIFT	23,316	24,617	23,509
CDS	CODON_INSERTION	1228	1321	1267
CDS	EXON_DELETED	1	1	1
CDS	CODON_DELETION	1364	1432	1390
CDS	CODON_CHANGE_PLUS_CODON_DELETION	1115	1151	1109
CDS	CODON_CHANGE_PLUS_CODON_INSERTION	423	440	426
CDS	STOP_GAINED	150	149	169
CDS	STOP_LOST	63	66	63

### Phylogenetic tree and PCA

**Fig. 2:**
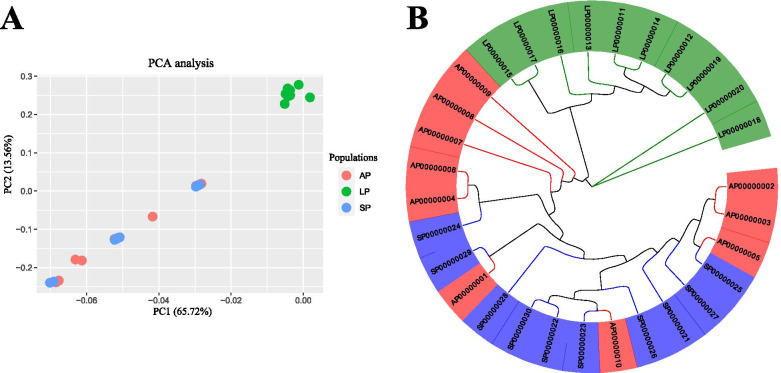
Principal component analysis (PCA) (A) and phylogenetic tree analysis (B)

PCA displayed that three populations (10 individuals per population) were clustered into two categories (Fig. 2A). One category is AP and SP, another is LP. Furthermore, distribution of AP and SP were scattered. Phylogenetic tree showed that AP was closely related to SP, while LP was distantly related to AP and SP (Fig. 2B).

### Analysis of selective sweeping

**Fig. 3:**
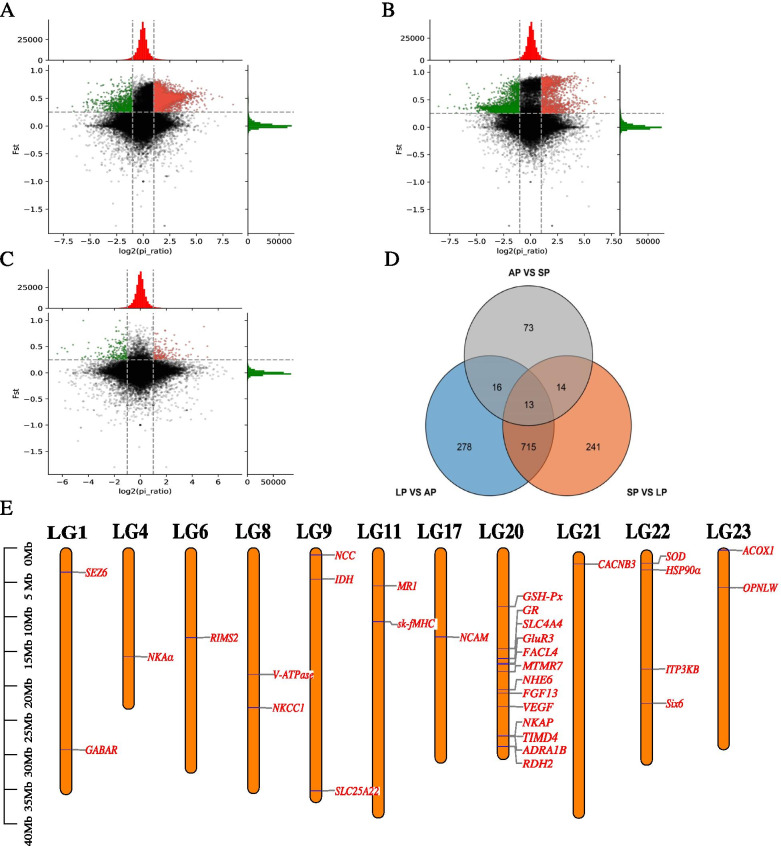
Results of genome-wide selective sweeping. (A) Selective sweeping result of AP vs LP. (B) Selective sweeping result of SP vs LP. (C) Selective sweeping result of AP vs SP. (D) venn diagram of selected genes numbers. (E) Chromosome location of selected genes related to immune, vision, migration, and osmoregulation

Under the condition of Fst > 0.25 and Pi ratio > 2 or Pi ratio < 0.5, 1022 genes were selected in AP vs LP (Fig. 3A). 983 genes were selected in LP vs SP (Fig. 3B). 116 genes were selected in AP vs SP (Fig. 3C). 13 genes were found in all three groups (Fig. 3D). After removing duplicates, 1224 genes were enriched on GO and KEGG, which were shown in Additional files 9 and 10: Fig. S4 and Fig. S5, respectively. Among these selected genes, the genes related to immune, vision, migration, and osmoregulation were identified. Chromosome locations of them were shown in Fig. 3E, mapped via TBtools [17].

### Activities of superoxide dismutase (SOD), glutathione peroxidase (GSH-Px), and glutathione reductase (GR) in livers of AP, LP, and SP

**Fig. 4:**
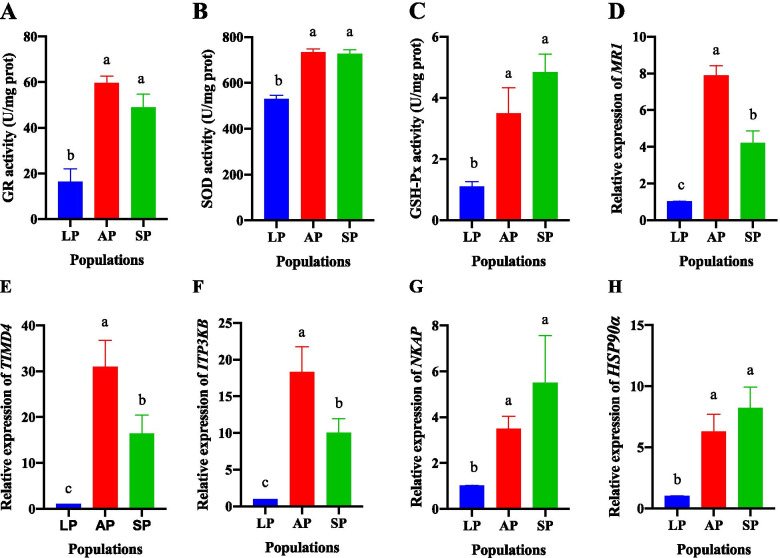
Detection of selected genes related to immune and oxidant stress in livers of AP, LP, and SP. (A-C) activities of GR, SOD, and GSH-Px. (D-H) expression of genes related to immune. The results were showed in means ± SD. Different letters indicate significant difference at *P* < 0.05

According to selective sweeping, genes related to oxidant stress were identified (*SOD*, *GSH-Px*, and *GR*) in AP vs LP and LP vs SP. GR activities in AP and SP were significantly higher than in LP (*P* < 0.05) (Fig. 4A). SOD and GSH-Px displayed similar trends (Fig. 4B-C).

### Expression of selected genes related to immune in livers of AP, LP, and SP

According to selective sweeping, genes related to immune were identified in AP vs LP and LP vs SP, including major histocompatibility complex class I-related gene protein (*MR1*), T-cell immunoglobulin and mucin domain-containing protein 4 (*TIMD4*), inositol-trisphosphate 3-kinase B (*ITP3KB*), NF-kappa-B-activating protein (*NKAP*), and heat shock protein HSP 90-alpha (*HSP90α*). Their expression in AP and SP was significantly higher than in LP (*P* < 0.05) (Fig. 4D-H).

### Expression of selected genes related to vision and rhythm in AP, LP, and SP

**Fig. 5:**
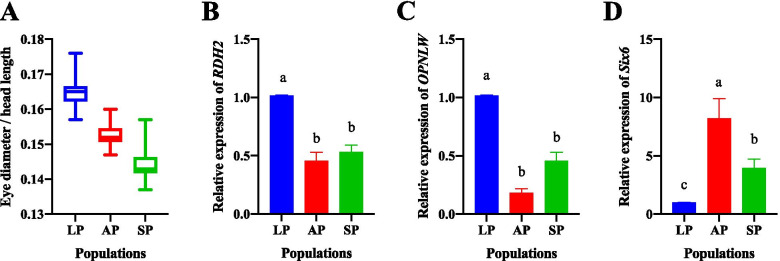
Detection of selected genes related to vision and rhythm in AP, LP, and SP. (A) eye diameter/head length. (B-C) expression of genes related to vision in eyes. (D) expression of gene related to rhythm in brains. The results were showed in means ± SD. Different letters indicate significant difference at *P* < 0.05

Eye diameter/head length was calculated in AP, LP, and SP. Eye diameter/head length of LP was bigger than AP and SP, but there was no significance among AP, LP, and SP (*P* > 0.05) (Fig. 5A). According to selective sweeping, genes related to vision and rhythm were identified in AP vs LP and LP vs SP, including Retinal dehydrogenase 2 (*RDH2*), long/medium wavelength-sensitive opsin (*OPNLW*), and Sine oculis homeobox protein 6 (*Six6*). Expression of *RDH2* and *OPNLW* in eyes of LP was significantly higher than AP and SP (*P* < 0.05) (Fig. 5B-C), while expression of *Six6* in the brain of LP was significantly higher than AP and SP (*P* < 0.05) (Fig. 5D).

### Expression of selected genes related to nerve conduction and long-distance migration in AP and LP

**Fig. 6:**
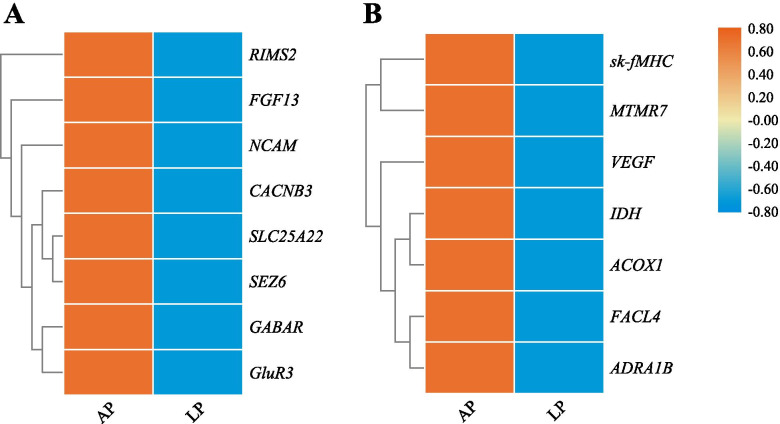
Detection of selected genes related to nerve conduction and long-distance migration in AP and LP. (A) expression of genes related to nerve conduction in brains. (B) expression of genes related to long-distance migration in muscles

According to selective sweeping, genes related to nerve conduction were identified in AP vs LP, including mitochondrial glutamate carrier 1 (*SLC25A22*), gamma-aminobutyric acid receptor (*GABAR*), glutamate receptor 3 (*GluR3*), seizure protein 6 (*SEZ6*), neural cell adhesion molecule (*NCAM*), regulating synaptic membrane exocytosis protein 2 (*RIMS2*), fibroblast growth factor 13 (*FGF13*), and voltage-dependent L-type calcium channel subunit beta-3 (*CACNB3*). Their expression in brains of AP was significantly higher than LP (*P* < 0.05) (Fig. 6A). Moreover, genes related to long-distance migration were identified in AP vs LP, including isocitrate dehydrogenase (*IDH*), peroxisomal acyl-coenzyme A oxidase 1 (*ACOX1*), long-chain fatty acid-CoA ligase type 4 (*FACL4*), adrenergic receptor alpha-1B (*ADRA1B*), vascular endothelial growth factor (*VEGF*), myosin heavy chain, fast skeletal muscle (*sk-fMHC*), and myotubularin related protein 7 (*MTMR7*). Their expression in muscles of AP was significantly higher than in LP (*P* < 0.05) (Fig. 6B).

### Expression of selected genes related to osmoregulation in gills of LP and SP

**Fig. 7:**
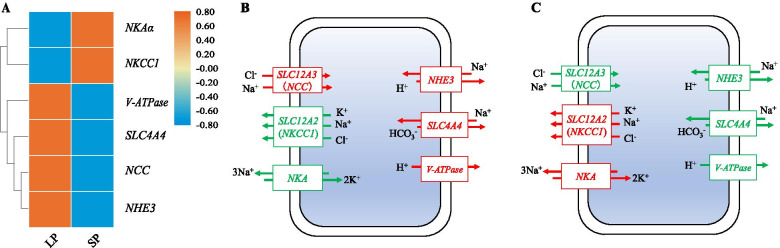
Detection of selected genes related to osmoregulation in LP and SP. (A) expression of genes related to osmoregulation in gills. (B) Osmoregulatory mechanisms of LP during hypotonic environment. (C) Osmoregulatory mechanisms of SP during hypertonic environment. Up regulated gene with italic were shown in red and boxed in red. Down regulated gene with italic were shown in green and boxed in green. The red arrows mean promotion, and the green arrows mean inhibition

According to selective sweeping, genes related to nerve conduction were identified in LP and SP, including sodium bicarbonate transporter (*SLC4A4*), Na^+^/Cl^−^ cotransporter (*NCC*), Na^+^/K^+^/2Cl^−^ cotransporter (*NKCC1*), sodium/hydrogen exchanger 6 (*NHE6*), V-type H^+^-transporting ATPase (*V-ATPase*), and sodium/potassium-transporting ATPase subunit alpha (*NKAα*). The expression of *NKAα* and *NKCC1* in LP was significantly lower than SP (*P* < 0.05), while expression of *NCC*, *SLC4A4*, *NHE6*, and *V-ATPase* in LP were significantly higher than SP (*P* < 0.05) (Fig. 7A). Osmoregulatory mechanisms of LP and SP during hypotonic environment (Fig. 7B) and hypertonic environment (Fig. 7C) were mapped.

## Discussion

Grounded in life histories of *C. nasus*, they were divided into three populations, AP, LP, SP. It is commonly assumed that these populations are different species, though they cannot be distinguished by outward. Conversely, it has been justified that these populations are the same species via amplified fragment length polymorphism (AFLP) markers [2]. In the present study, PCA and phylogenetic analysis showed that three populations were clustered into two categories. One category is AP and SP, another is LP. Nevertheless, AP was closely related to SP. Our results were consistent with the concept demonstrated by Liu et al. [3]. LP has been locked in the lake for a long time because of multifaceted reasons. We speculated that as time goes on, the farther genetic distance between AP and LP was attributed to different living environments. The closer genetic distance between AP and SP was attributed to genetic mutation.

### Immune and oxidant stress

Because of their different life histories, AP and SP live in the marine, but LP lives in freshwater lake. AP and SP are highly susceptible to be infected with Anisakidae parasites, a kind of parasites living in marine only [18, 19]. Parasite infection will trigger oxidant stress and immune responses. In the present study, *SOD*, *GSH-Px*, and *GR* were selected, and activities of these enzymes in AP and SP were significantly higher than in LP. SOD, GSH-Px, and GR play critical roles in removing active oxygen free radicals and repairing damaged cells. Moreover, *MR1*, *TIMD4*, *ITP3KB*, *NKAP*, and *HSP90α* were selected, and expression of these genes in AP and SP were significantly higher than in LP. MR1 is associated with antigen processing and interaction with T cells [20, 21]. TIMD4 involves in regulating T-cell proliferation and lymphotoxin signaling [22]. ITP3KB participates in signal transduction and activation of immune cell activation [23]. NKAP plays a role as a transcriptional corepressor of the Notch-mediated signaling required for T-cell development and involves in the TNF and IL-1 induced NF-kappa-B activation [24, 25]. HSP90α can mediate antigen processing and presentation via major histocompatibility complex class I antigen processing pathway [26]. Transcriptomic analysis revealed that parasite infection could activated antigen processing and presentation and initiate the T cell receptor signaling pathway in migratory *C. nasus* [19], which was like our results. Hence, these selected genes may play a vital role in increasing immunity of anadromous population and sea population response to parasite infection.

### Vision and rhythm

Fish vision is an important sense to receive environmental information, which plays key roles in feeding, courtship, information transmission, and evasion from enemies [27]. Genes related to vision are critical for evolution and species formation. Fish need to improve their visual system to adapt to the constant changes of the external light environment. In the present study, *RDH2* was selected, and its expression in AP and SP was significantly lower than in LP. RDH2 converts retinaldehyde to all-trans-retinoic acid (atRA) in response to visual signals [28]. And it can regulate postnatal ocular growth in humans through the synthesis of atRA [29]. Eye diameter/head length of landlocked population was bigger than anadromous population and sea population. Anadromous population and sea population was living in the continental shelf of the East China Sea whose water depth was < 60 m [2]. However, landlocked population was living in freshwater Taihu whose visibility was low. Under normal circumstances, the pupils of fish will not dilate and shrink. Therefore, Fish need to increase the eye diameter to ensure the amount of light. Moreover, *OPNLW* was selected, and its expression in AP and SP were significantly lower than in LP. OPNLW is red-sensitive opsin and light-absorbing molecules that mediate vision [30]. Suspended particles in freshwater can cause short-wavelength light scattering, which may cause the visual image of fish to fade. Freshwater quality is very permeable to red light, so freshwater fish usually have blue and green opsins, as well as red opsins (trichromats), which may be more beneficial for fish to survive in freshwater [31]. *Six6* was selected, and its expression in AP and SP was significantly higher than in LP. Six6 is required for early eye development, GnRH neuron, and SCN development in vertebrates [32, 33]. The suprachiasmatic nucleus (SCN), the brain’s primary circadian pacemaker, is required to translate day-length and circadian rhythms into neuronal, hormonal, and behavioral rhythms [34]. Migration is a rhythmic behavior. Six6 mutation may affect migration via disordering rhythm in landlocked *C. nasus*. Thus, these selected genes were expected to play important roles in vision and rhythm.

### Nerve conduction and long-distance migration

In the present study, *SLC25A22*, *GABAR*, *GluR3*, *SEZ6*, *NCAM*, *RIMS2*, *FGF13*, and *CACNB3* were selected, and their expression in AP was significantly higher than in LP. SLC25A22 is involved in the transport of glutamate (excitatory neurotransmitter) across the inner mitochondrial membrane [35, 36]. Gamma-aminobutyric acid (GABA) is the main inhibitory neurotransmitter in the brain. GluR3 that functions as a ligand-gated ion channel in the central nervous system and plays an important role in excitatory synaptic transmission [37]. SEZ6 is required for the development and maintenance of the nervous system. May play a role in cell-cell recognition and neuronal membrane signaling. Involved in the development of appropriate excitatory synaptic connectivity [38]. NCAM has been implicated as having a role in cell-cell adhesion, neurite outgrowth, synaptic plasticity, and learning and memory [39]. RIMS2 plays a role in dendrite formation by melanocytes [40]. FGF13 participates in the refinement of axons by negatively regulating axonal and leading processes branching and plays a crucial role in neuron polarization and migration in the cerebral cortex and the hippocampus [41]. It may regulate voltage-gated sodium channel transport and function and is required for the development of axonal initial segment-targeting inhibitory GABAergic synapses made by chandelier neurons [42]. CACNB3 was strongly upregulated in distinct dendritic cell populations upon stimulation [43]. Similar to our results, genes bound up with neuronal signaling and sensory system were up-regulated in *C. nasus* based on transcriptomic analysis [15, 16]. Previous researches have been proved that visual and olfactory systems play essential roles in migration in anadromous fish [44, 45]. These findings suggested that nerve conduction might be bound up with migration, and these selected genes could be considered as major genes of controlling migration.

At the beginning of spring, the sexually mature fish run thousands of kilometers from marine to river to spawn. However, they generally do not feed during spawning migration, which requires lots of energy and high athletic capacity. In the present study, *IDH*, *ACOX1*, *FACL4*, *ADRA1B*, *VEGF*, *sk-fMHC*, and *MTMR7* were selected, and expression of them in AP was significantly higher than in LP. IDH is the rate-limiting enzyme in the tricarboxylic acid cycle involved in cell energy metabolism, catalyze the oxidative decarboxylation of isocitrate to 2-oxoglutarate [46]. ACOX1 catalyzes the desaturation of acyl-CoAs to 2-trans-enoyl-CoAs, which is the first enzyme of the fatty acid beta-oxidation pathway [47]. FACL4 is a key enzyme involved in the metabolism of AA, EPA, and DHA [48]. ADRA1B is distributed on the presynaptic membrane and vascular smooth muscle, and it mainly causes vasoconstriction when excited [49]. VEGF is a highly specific vascular endothelial cell growth factor that promotes vascular permeability, extracellular matrix degeneration, vascular endothelial cell migration, proliferation, and angiogenesis, etc. [50]. sk-fMHC functions on muscle contraction [51, 52]. MTMR7 is involved in myogenesis [53]. Genes related to cardiovascular, hematopoietic, digestion, and metabolism played critical roles in long-term migration via selective sweeping and transcriptomic analysis in migratory *C. nasus* [54]. These selected genes associated with metabolism and blood vessels were also found in our study, which may contribute to highly efficient long-term migration.

### Osmoregulation

Because of their different life histories, sea population is living in the hypertonic environment, while landlocked population is living in hypotonic environment. Their osmoregulatory mechanisms are different. In the present study, *NKAα*, *NKCC1*, *NCC*, *SLC4A4*, *NHE6*, and *V-ATPase* were selected. Most euryhaline bony fishes can regulate the activity of NKA to adapt to salinity changes of the external environment. Its main function is to transport 3 Na^+^ out of the cell and 2 K^+^ into the cell at the same time [55]. NCC and NKCC1 are electrically neutral ion transporters. NCC involves in the tranport of Na^+^ and Cl^−^ from extra-cellular to intra-cellular [56, 57], while NKCC1 participates in the transport of Na^+^, K^+^, and Cl^−^ from intra-cellular to extra-cellular [58]. The exchange of intracellular H^+^ and extracellular Na^+.^ is depended on NHE3 [59, 60]. The expression levels of *NKAα* and *NKCC1* in LP were lower than SP, while expression levels of *NCC* and *NHE3* in LP were higher than SP. Up-regulated expression of *NCC* in *O. mossambicus* gills under hypotonic stress can enhance absorption of Na^+^ and Cl^−^ to augment plasma osmotic pressure [56]. Conversely, increased expression of *NKCC1* was in *Salvelinus namaycush*, *Salvelinus fontinalis*, and *Salmo salar* gills under hypertonic stress can promote ion secretion to reduce plasma osmotic pressure [55]. In contrast, decreased expression was displayed in *Dicentrarchus labrax* gills under hypertonic stress [61]. Moreover, intercellular pH homeostasis will be affected by salinity changes. V-ATPase is responsible for H^+^ transport [62]. SLC4A4 plays indispensable roles in acid-base homeostasis via pumping HCO_3_^−^ [63]. The expression levels of *SLC4A4* and *V-ATPase* in LP were higher than SP. Our results indicated that their expression would be beneficial to maintain pH stability in gills of LP and SP. Besides maintaining pH stability, V-ATPase can also generate a potential difference between the inside and outside of the membrane by pumping out H^+^, which will promote to transport Na^+^ from extra-cellular to inter-cellular through NHE [64]. Therefore, we speculated that these selected genes would play central parts in osmoregulation.

## Conclusion

In summary, we performed whole-genome resequencing of three *C. nasus* populations, anadromous population, landlocked population, and sea population. Single nucleotide polymorphisms (SNPs) and insertions and deletions (InDels) were identified and annotated. Then, selected genes related to immune, vision, migration, and osmoregulation were identified via selective sweeping. These genes were detected via qRT-PCR, and their expression displayed significant differences among three *C. nasus* populations. Our findings reveal molecular mechanism of differences in immune, vision, migration, and osmoregulation among different *Coilia nasus* populations, and will provide valuable resources for aquaculture and protection on *C. nasus*.

## Methods and materials

### Sample collection

*C. nasus* used in this study were sampled in the Yangtze River (N 32.2274, E 119.3643), Lake Taihu (N 31.3271, E 120.0245), and East China Sea (N 31.8646, E 122.5728). According to Yang et al., 2006 [65], Sr/Ca of otolith fingerprint element technology was performed to classify anadromous population (AP), landlocked population (LP), and sea population (SP). The detail information of them were shown in Table 3. The sampled fish were anesthetized via 70 mg/L buffered tricaine methanesulfonate (MS-222) (Greenhengxing, Beijing, China). 10 individuals of each population were selected, and their eyes, gills, livers, brains, muscles were sampled and put in liquid nitrogen immediately, then stored at − 80 °C until using.

**Table 3:** Sampling details of different *C. nasus* populations

Population	Sample code	Body length (cm)	Body weight (g)	Age	Sexual maturity	Sr/Ca × 10^3^
Anadromous population	AP1	23.4	75.1	3	♀II	5.2
	AP2	25.8	76.5	2	♀III	5.4
	AP3	24.6	71.2	3	♀II	4.7
	AP4	25.9	76.3	2	♀II	6.1
	AP5	26.1	82.1	2	♀III	4.9
	AP6	27.8	79.5	2	♀III	5.3
	AP7	30.1	100.3	3	♀II	4.4
	AP8	22.8	71.3	2	♂III	4.9
	AP9	24.8	72.6	3	♀III	6.1
	AP10	30.2	106.2	2	♀IV	4.2
Landlocked population	LP1	12.3	30.3	1	N	1.9
	LP2	13.7	29.6	1	N	2
	LP3	11.6	27.3	1	N	2.4
	LP4	9.7	19.7	1	N	2.3
	LP5	16.5	31.3	1	N	1.8
	LP6	14.3	28.7	1	N	2.6
	LP7	13.6	25.5	1	N	2.2
	LP8	12.6	21.5	1	N	1.7
	LP9	15.8	32.6	1	N	1.7
	LP10	10.9	23.1	1	N	2.1
Sea population	SP1	22.3	59.4	3	♀III	7.9
	SP2	23.9	91.5	2	♀III	8
	SP3	21.6	84.7	2	♀II	8.1
	SP4	24.2	94.5	3	♀II	7.7
	SP5	34.2	136.1	3	♀IV	8.4
	SP6	30.9	99.8	3	♀II	7.5
	SP7	34.4	140.4	2	♀IV	7.6
	SP8	27.6	108.4	3	♀III	8
	SP9	35.9	159.2	2	♀IV	7.9
	SP10	25.9	84.3	2	♀II	7.3

“N” represents “not sexually mature”

### DNA isolation and genome resequencing

Muscle tissues (10 individuals of each population) were used to extract genome DNA used Qiagen Genomic Tip100 (Qiagen, Hilden, Germany). After the genomic DNA of the sample is qualified, the DNA (3.0 μg DNA from each individual) was fragmented by mechanical interruption method (ultrasound), and then the fragmented DNA was purified, end-repaired, added with A at the 3′ end, connected to the sequencing adapter, and then applied to agarose gel electrophoresis was used for fragment size selection, PCR amplification was performed to form a sequencing library, the built library was first subjected to library quality inspection, and the library that has passed the quality inspection was sequenced by Illumina 2500 platform. After filtering adaptors, ambiguous “N” nucleotides, and low-quality sequences, clean reads were generated for further analysis.

### Detection and annotation of SNPs and InDels

Generated clean reads were mapped on *C. nasus* reference genome (PRJNA421870) using BWA [66]. According to location of clean reads on reference genome, Picard was used to filter redundant reads (MarkDuplicates) to ensure the accuracy of the detection results. Then GATK’s HaplotypeCaller (local haplotype assembly) algorithm was used for SNP and InDel mutation detection [67]. After filtering, the mutation sites were generated. The main filtering parameters were as follows: (1) Based on the subroutine vcfutils.pl (varFilter -w 5 -W 10) in bcftools to filter out SNPs within 5 bp of INDEL and adjacent INDEL within 10 bp; (2) clusterSize 2 clusterWindowSize 5, which meant that the number of mutations in a 5 bp window should not exceed 2; (3) QUAL < 30, the quality value in Phred format, indicating the possibility of variant mutation at this site. If the quality value was lower than 30, it would be filtered out; (4) QD < 2.0, the ratio of the variation quality value (Quality) divided by the coverage depth (Depth), the coverage depth was the sum of the coverage depths of all samples containing variant bases at this site. Those with QD lower than 2.0 were filtered out; (5) MQ < 40, the root mean square of the alignment quality values of all reads aligned to this site. Filter out those with MQ lower than 40; (6) FS > 60.0, the value converted from the *p*-value of Fisher’s test, which describes whether there was a significant positive or negative for the read containing only mutations and the read containing only the base of the reference sequence during sequencing or comparison Chain specificity. In other words, there would be no chain-specific comparison results, and FS should be close to zero. Filter out those with FS higher than 60; (7) Other mutation filtering parameters were processed by default values officially designated by GATK. SNPs and InDels were annotated via SnpEff [68]. According to the location of the mutation site on the reference genome and the gene location information on the reference genome, the region where the mutation site occurs in the genome (intergenic region, gene region or CDS region, etc.), and the impact of the mutation (synonymous, non-synonymous mutations, etc.).

### Phylogenetic tree analysis and principal component analysis (PCA)

The generated vcf file (SNPs information) was transformed into phylip file via vcf2phylip (open-source code from Github website). The generated phylip file was used to construct neighbor joining phylogenetic tree (bootstrap 1000) via MEGAX software. Then, the phylogenetic tree was beautified via evolview (https://evolgenius.info//evolview-v2/) [69]. The principal components were calculated via PLINK 1.9, then visualization of the generated result was displayed via Rsudio package ggplot2.

### Genome-wide selective sweep test

The polymorphism levels were quantified by pairwise nucleotide diversity *θ*_*π*_ (Pi). The genetic differentiation was quantified by pairwise Fst. Both Pi and Fst were calculated by the sliding window method (100 kb windows sliding in 10 kb steps) [54]. Regions with Fst > 0.25 and Pi ratio > 2 or Pi ratio < 0.5 were identified as selected regions [54]. Genes in the selected regions were identified via bedtools. These genes were annotated on NR, swiss-prot, KOG, PFAM, GO, and KEGG databases via BLAST. GO and KEGG enrichment analysis was performed using the OmicShare tools, a free online platform for data analysis (http://www.omicshare.com/tools) [70, 71].

### Detection of antioxidants activity in liver

Liver tissue stored at − 80 °C was weighed accurately. The liver tissue (approximately 0.1 g) was homogenized in nine volumes of normal saline. The homogenate was centrifuged at 250 r/min for 10 min. Then the supernatant (10% tissue homogenate) was taken for further analysis. The activities of superoxide dismutase (SOD), glutathione peroxidase (GSH-Px), and glutathione reductase (GR) were detected by kits, according to their manufacturers’ instructions (Jiancheng, Nanjing, China), respectively.

### Gene expression analysis via qRT-PCR

RNA of eyes, brains, gills, livers, and muscles in AP, LP, and SP (5 individuals of each population) were extracted. Liver RNA was used to detect expression levels of genes related to immune. Brain RNA was used to detect expression levels of genes associated with rhythm and nerve conduction. Eye RNA was used to detect expression levels of genes bound up with vision. Muscle RNA was used to detect expression levels of genes associated with long-distance migration. Gill RNA was used to detect expression levels of genes bound up with osmoregulation. The details of detection of the qualities of extracted RNA were described in Gao et al. (2021) [72]. The first strand cDNA was synthesized according to the manufacturer’s instruction of HiFiScript cDNA Removal RT MasterMix (Cowin Biosciences, Taizhou, China). Primer Premier 5 software was used to design the primers used for qRT-PCR (Additional file 11: Table S6). The coding sequences were from reference genome (PRJNA421870). The qRT-PCR program was set as described in Gao et al. (2020a, 2020b) [73, 74]. Expression of selected genes was normalized by the geometric mean of *β-actin*, *18SrRNA*, and *GAPDH*, housekeeper genes [75]. All samples were analyzed in triplicate and the expression of target genes was calculated using the 2^-ΔΔCT^ method.

### Statistical analysis

To determine significant differences among three populations, one-way analysis of variance and Duncan test was performed via SPSS 20 software. *t*-test was performed via SPSS 20 software to determine significant differences between two populations. All data were shown as mean ± SD. *P <* 0.05 indicated significant differences. Histograms were mapped via GraphPad 8.0, and heatmaps were mapped via TBtools [17].

## Supplementary Information

**Additional file 1.**

**Additional file 2.**

**Additional file 3.**

**Additional file 4.**

**Additional file 5.**

**Additional file 6.**

**Additional file 7.**

**Additional file 8.**

**Additional file 9.**

**Additional file 10.**

**Additional file 11.**

## Data Availability

The dataset supporting the conclusions of this article is available in the NCBI Sequence Read Archive (SRA) repository, accession number PRJNA744015 (https://www.ncbi.nlm.nih.gov/bioproject/PRJNA744015).
