# Supplementary material for: Whole-genome resequencing of three Coilia nasus population reveals genetic variations in genes related to immune, vision, migration, and osmoregulation
Source: BMC Genomics. 2021 Dec 6;22:878. doi: 10.1186/s12864-021-08182-0 (PMC8647404; doi:10.1186/s12864-021-08182-0)
Supplement: Supplementary file 1 — Additional file 1. [file 12864_2021_8182_MOESM1_ESM.docx]

Table S1. Statistics of the whole genome resequencing and mapping rate.

|  | Clean_Reads | Clean_Base | Q20(%) | Q30(%) | GC(%) | Mapped(%) |
| --- | --- | --- | --- | --- | --- | --- |
| AP | 296613598 | 88836041388 | 95.297 | 89.711 | 43.907 | 95.098 |
| LP | 327203910 | 97967399734 | 95.24 | 89.913 | 44.031 | 95.37 |
| SP | 300660251 | 89977946222 | 95.743 | 90.944 | 44.075 | 95.377 |
