# Supplementary material for: Whole-genome resequencing of three Coilia nasus population reveals genetic variations in genes related to immune, vision, migration, and osmoregulation
Source: BMC Genomics. 2021 Dec 6;22:878. doi: 10.1186/s12864-021-08182-0 (PMC8647404; doi:10.1186/s12864-021-08182-0)
Supplement: Supplementary file 2 — Additional file 2. [file 12864_2021_8182_MOESM2_ESM.docx]

Table S2. Statistics of SNP types.

| Sample | SNP number | Transition | Transversion | Ti/Tv | Heterozygosity | Homozygosity | Het-ratio |
| --- | --- | --- | --- | --- | --- | --- | --- |
| AP | 3176204 | 1783585 | 1392619 | 1.28 | 968651 | 2207553 | 30.49% |
| LP | 3307069 | 1855653 | 1451416 | 1.27 | 965564 | 2341505 | 29.19% |
| SP | 3207906 | 1800730 | 1407176 | 1.27 | 988916 | 2218990 | 30.82% |
