# Supplementary material for: Whole-genome resequencing of three Coilia nasus population reveals genetic variations in genes related to immune, vision, migration, and osmoregulation
Source: BMC Genomics. 2021 Dec 6;22:878. doi: 10.1186/s12864-021-08182-0 (PMC8647404; doi:10.1186/s12864-021-08182-0)
Supplement: Supplementary file 3 — Additional file 3. [file 12864_2021_8182_MOESM3_ESM.docx]

Table S3. Statistics of InDels types.

| Sample | CDS-Insertion | CDS-Deletion | CDS-Homo | CDS-Het | CDS-Total | Genome-Insertion | Genome-Deletion | Genome-Homo | Genome-Het | Genome-Total |
| --- | --- | --- | --- | --- | --- | --- | --- | --- | --- | --- |
| AP | 15575 | 14216 | 27010 | 2781 | 29791 | 980515 | 881762 | 1526694 | 335583 | 1862277 |
| LP | 16516 | 14922 | 28398 | 3040 | 31438 | 1039017 | 932457 | 1623657 | 347817 | 1971474 |
| SP | 15776 | 14297 | 27006 | 3067 | 30073 | 995702 | 896393 | 1544736 | 347359 | 1892095 |
