# Supplementary material for: Whole-genome resequencing of three Coilia nasus population reveals genetic variations in genes related to immune, vision, migration, and osmoregulation
Source: BMC Genomics. 2021 Dec 6;22:878. doi: 10.1186/s12864-021-08182-0 (PMC8647404; doi:10.1186/s12864-021-08182-0)
Supplement: Supplementary file 4 — Additional file 4. [file 12864_2021_8182_MOESM4_ESM.docx]

Table S4. Length distribution of InDels on genome.

|  | AP-INS | AP-DEL | LP-INS | LP-DEL | SP-INS | SP-DEL |
| --- | --- | --- | --- | --- | --- | --- |
| 1bp | 497626 | 386129 | 528957 | 409767 | 505822 | 392966 |
| 2bp | 131402 | 145962 | 139277 | 155022 | 133768 | 148750 |
| 3-6bp | 118741 | 157687 | 124930 | 165878 | 280894 | 160248 |
| 7-10bp | 33852 | 46794 | 35644 | 49139 | 194716 | 47411 |
| >10bp | 34577 | 57145 | 36384 | 59962 | 35097 | 58105 |
