# Supplementary material for: Whole-genome resequencing of three Coilia nasus population reveals genetic variations in genes related to immune, vision, migration, and osmoregulation
Source: BMC Genomics. 2021 Dec 6;22:878. doi: 10.1186/s12864-021-08182-0 (PMC8647404; doi:10.1186/s12864-021-08182-0)
Supplement: Supplementary file 5 — Additional file 5. [file 12864_2021_8182_MOESM5_ESM.docx]

Table S5. Length distribution of InDels on CDS.

|  | AP-INS | AP-DEL | LP-INS | LP-DEL | SP-INS | SP-DEL |
| --- | --- | --- | --- | --- | --- | --- |
| 1bp | 10074 | 7217 | 10607 | 7590 | 10095 | 7204 |
| 2bp | 1603 | 1953 | 1728 | 2088 | 1647 | 2020 |
| 3-6bp | 1743 | 2849 | 1854 | 2968 | 1826 | 2825 |
| 7-10bp | 321 | 646 | 349 | 667 | 327 | 685 |
| >10bp | 385 | 918 | 410 | 966 | 400 | 950 |
