# Supplementary figures and images for: Whole-genome resequencing of three Coilia nasus population reveals genetic variations in genes related to immune, vision, migration, and osmoregulation
Source: BMC Genomics. 2021 Dec 6;22:878. doi: 10.1186/s12864-021-08182-0 (PMC8647404; doi:10.1186/s12864-021-08182-0)

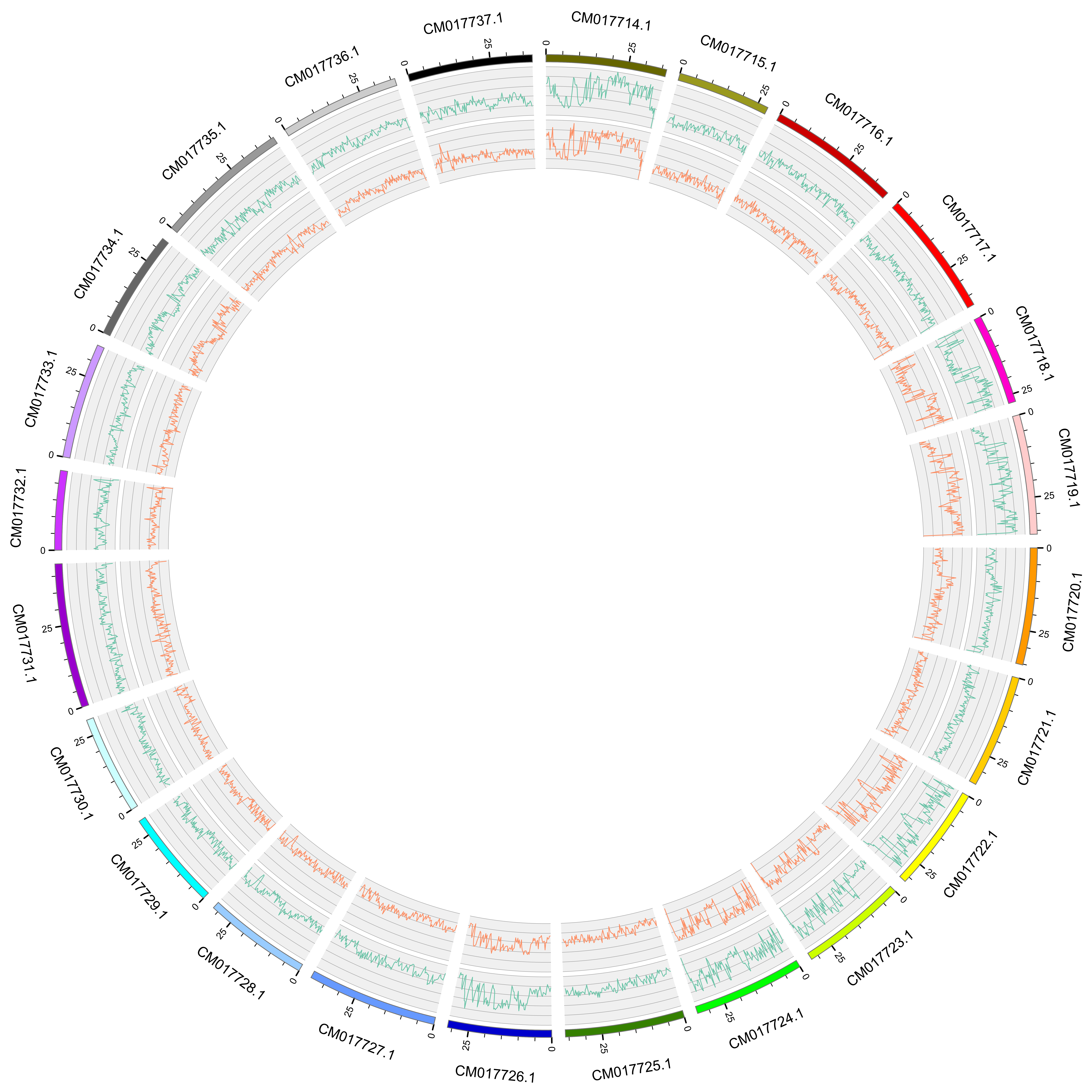

Supplement: Supplementary file 6 — Additional file 6. [file 12864_2021_8182_MOESM6_ESM.pdf]

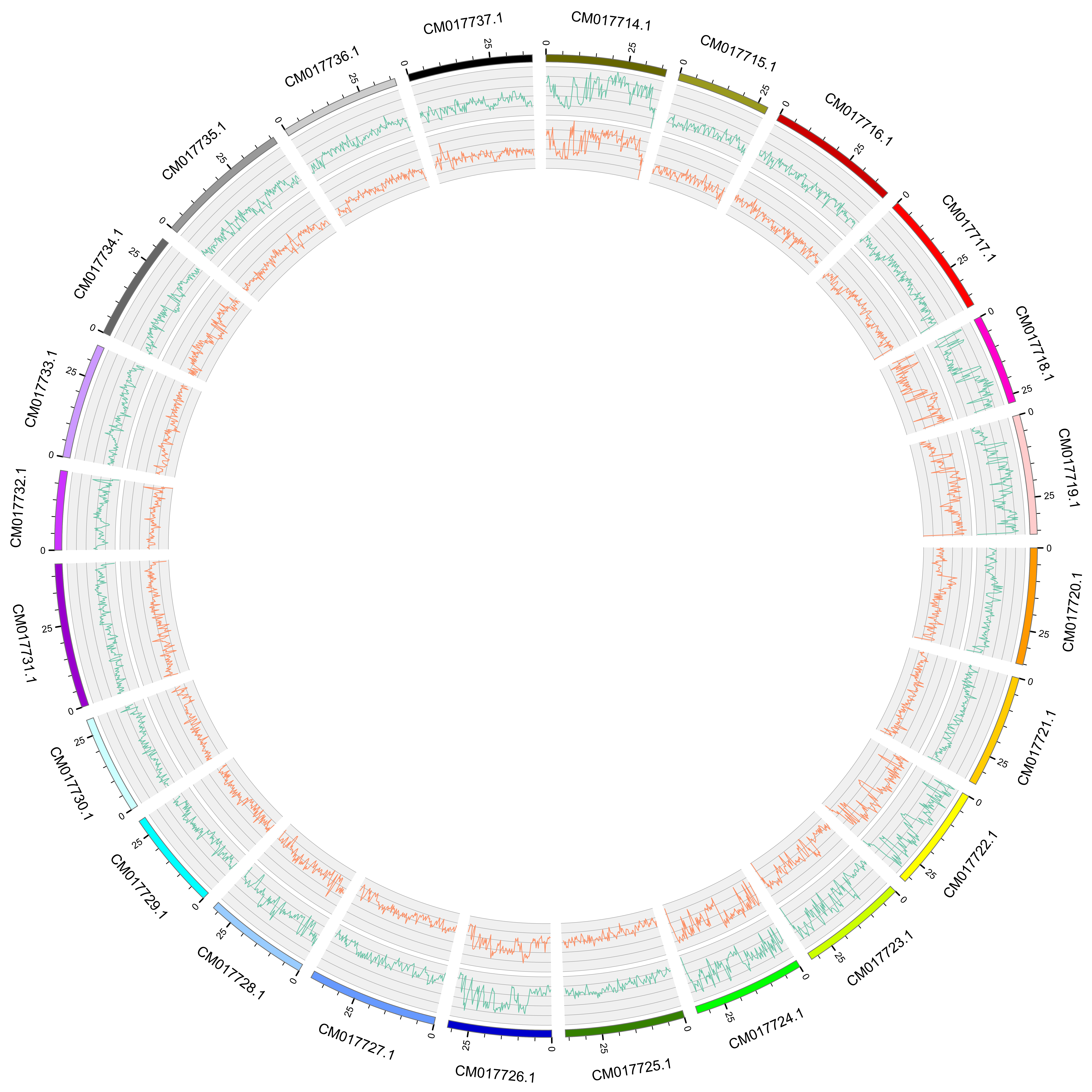

Supplement: Supplementary file 7 — Additional file 7. [file 12864_2021_8182_MOESM7_ESM.pdf]

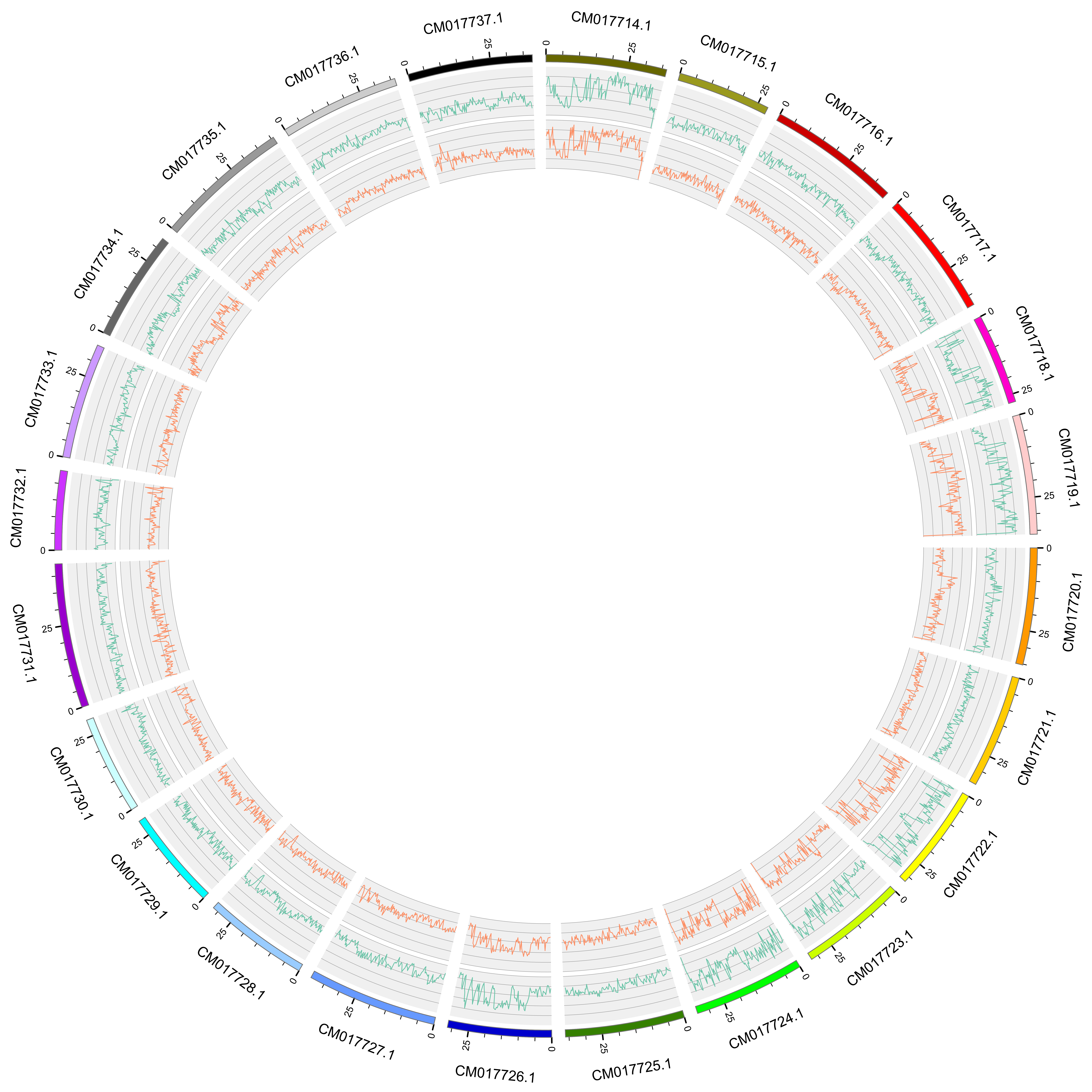

Supplement: Supplementary file 8 — Additional file 8. [file 12864_2021_8182_MOESM8_ESM.pdf]

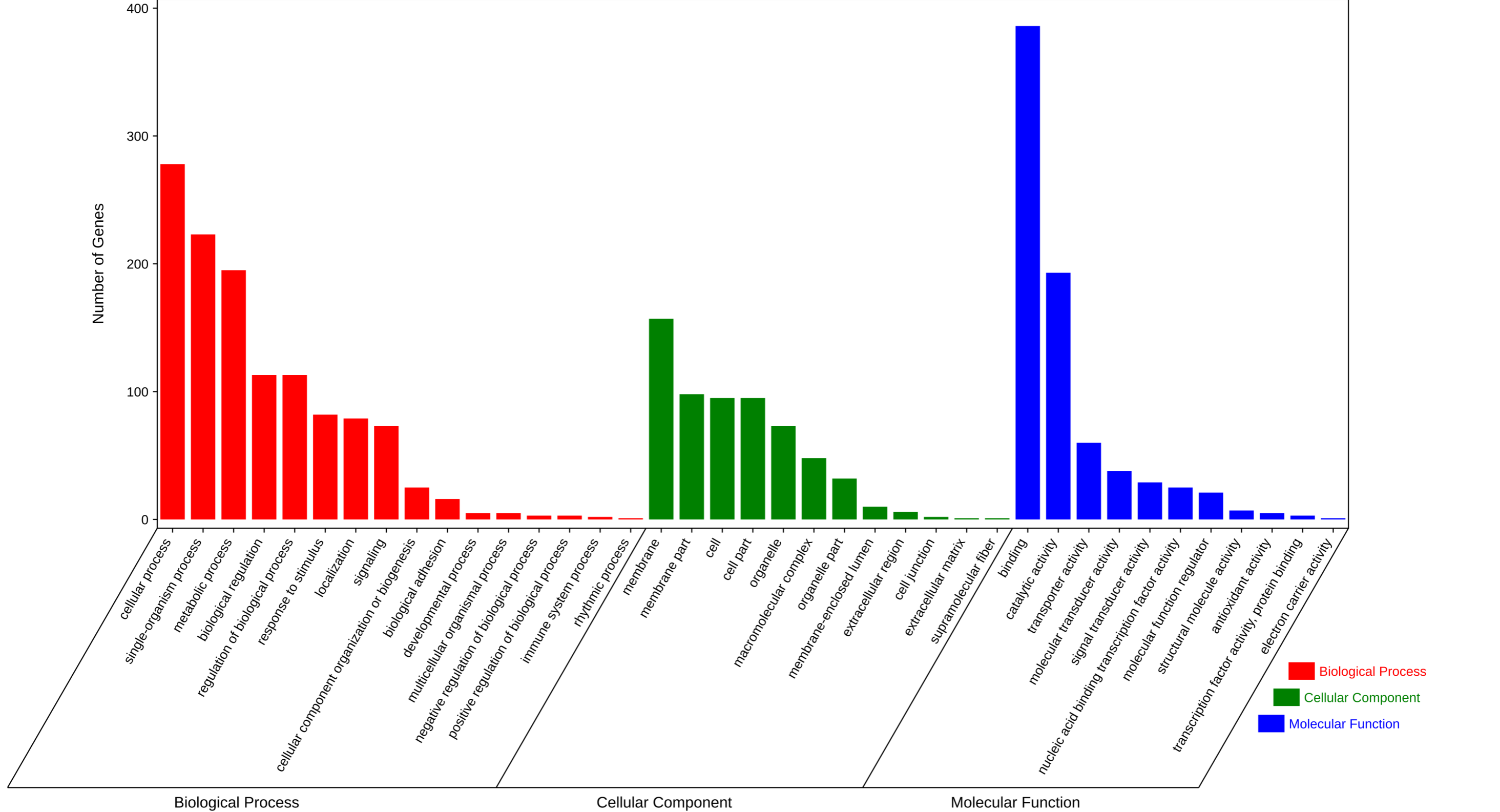

Supplement: Supplementary file 9 — Additional file 9. [file 12864_2021_8182_MOESM9_ESM.pdf]

# KEGG pathway annotation

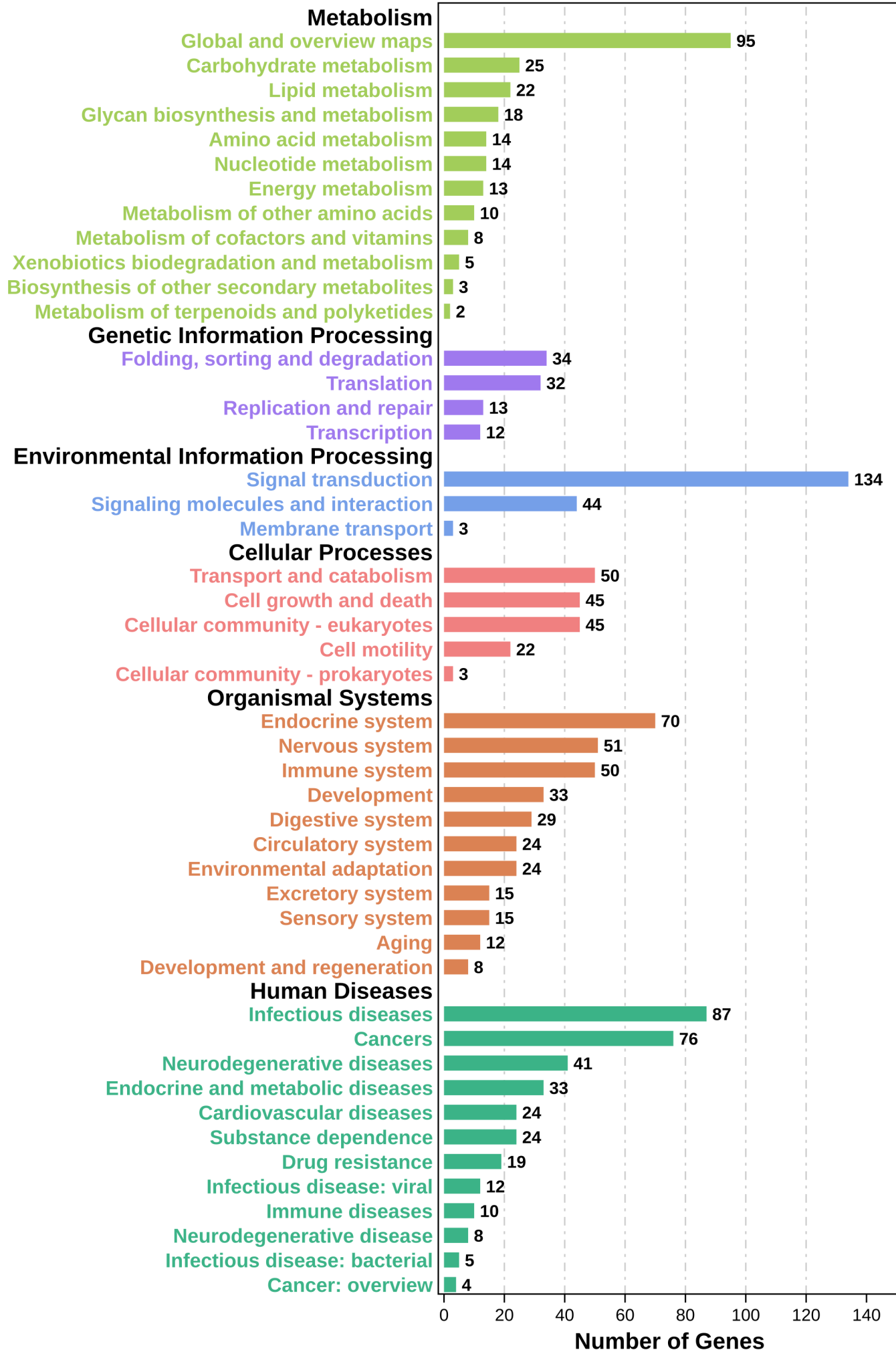

Supplement: Supplementary file 10 — Additional file 10. [file 12864_2021_8182_MOESM10_ESM.pdf]
