# Supplementary material for: Whole-genome resequencing of three Coilia nasus population reveals genetic variations in genes related to immune, vision, migration, and osmoregulation
Source: BMC Genomics. 2021 Dec 6;22:878. doi: 10.1186/s12864-021-08182-0 (PMC8647404; doi:10.1186/s12864-021-08182-0)
Supplement: Supplementary file 11 — Additional file 11. [file 12864_2021_8182_MOESM11_ESM.docx]

Table S6. Primers used for qRT-PCR in this study.

| Gene |  | Sequence (5’- 3’) | Product length (bp) |
| --- | --- | --- | --- |
| *β-actin* | Forward | AACGGATCCGGTATGTGCAAAGC | 110 |
|  | Reverse | GGGTCAGGATACCTCTCTTGCTCTG |  |
| *18S rRNA* | Forward | TGATTGGGACTGGGGATTGAA | 114 |
|  | Reverse | TAGCGACGGGCGGTGTGT |  |
| *GAPDH* | Forward | AGCTTGCCACCCTCTTGCT | 121 |
|  | Reverse | AGCCATCAACGACCCCTTC |  |
| *MR1* | Forward | AGACTGTGTTGAGTGGCTGA | 105 |
|  | Reverse | GGTCCGGTAGAGCCTTTCTG |  |
| *NKAP* | Forward | TCATCGCCCCGATAACGAAA | 200 |
|  | Reverse | TAGATCTGGAGCGACTGCGA |  |
| *TIMD4* | Forward | GCGGAAAACTCGAAGATGGG | 181 |
|  | Reverse | GGAACTACAGGGGTGGTGATG |  |
| *HSP90* | Forward | CATTATGTTCCCGGCCCTCT | 136 |
|  | Reverse | CTGGCTCTGTGGGTTTGACT |  |
| *ITP3KB* | Forward | CTCCCAGTGTTCCCAACCAG | 199 |
|  | Reverse | CTGCCCCTCTGCTCTGTATT |  |
| *OPNLW* | Forward | TAATAGGCGGATGTGGCGAG | 140 |
|  | Reverse | CGAGGTATGCGGTCCCTTTT |  |
| *RDH2* | Forward | CCTTCAAGACCCCTACCCGA | 136 |
|  | Reverse | TAACCTGTTTTTCGCGGCTG |  |
| *SIX6* | Forward | GACAAAGAGACAGAGCCGCA | 172 |
|  | Reverse | GTGATAGAGATGGCCGACGC |  |
| *NCC* | Forward | TCGGGCTTATCTTCGCCTTC | 189 |
|  | Reverse | CCCACTCCATTCCTGCCATT |  |
| *NKCC* | Forward | CACTGGGCTGTCTACGTCTG | 112 |
|  | Reverse | GAGCCCAATGGATCCACCAA |  |
| *NKA* | Forward | CAGAAACCCCAAGACCGACA | 181 |
|  | Reverse | GTTGTTGTTGCGATCGTCCC |  |
| *SCL4A4* | Forward | GTTGTTGTTGCGATCGTCCC | 127 |
|  | Reverse | TGTCACTCTTCCTGATGGCG |  |
| *V-ATPase* | Forward | TACGGAGCCTACCCTTCTGT | 103 |
|  | Reverse | AGCCTCGCAAATGTGGAAGT |  |
| *NHE3* | Forward | CGGGGCTTTCCTGGGTATTT | 157 |
|  | Reverse | GATGGACAGGGCAAAGGTCA |  |
| *IDH* | Forward | GATGGTCTTCTCTCCCGCTG | 106 |
|  | Reverse | CCTCACCTCGTCTGTGTTGT |  |
| *ACOX1* | Forward | ACATCATTGGCACCTACGCA | 181 |
|  | Reverse | GCCAGGACTACAGCATGGTT |  |
| *ADRA1B* | Forward | TCTTGAGGCGGGAGTTATGC | 116 |
|  | Reverse | CTGGATGCCCTTGACCTCTG |  |
| *FACL4* | Forward | AAAGACAAACCTCACCCCCG | 141 |
|  | Reverse | AACATCGCCTGTGCAAAACC |  |
| *VEGF* | Forward | AAAATCAGCCGTGCTCCACA | 118 |
|  | Reverse | GGGCTTGATCCTCCGGTTTT |  |
| *sk-fMHC* | Forward | GCCGACAACAGGACTTACGA | 122 |
|  | Reverse | AAGACTGCTGTGCCTTTGGT |  |
| *MTMR7* | Forward | AGACAAAGGCGAGGGTTCTG | 171 |
|  | Reverse | ACTGGTAGGGTGGCTGATCT |  |
| *SLC25A22* | Forward | CGACTGTGTCAGCGAAAAGC | 198 |
|  | Reverse | CGAACGACCTTGAGCGAGTA |  |
| *GABAR* | Forward | ATCAGTGCCAGAAACTCGCT | 139 |
|  | Reverse | AAGCGTACCCCCTCTTTGTG |  |
| *GluR* | Forward | AAGACACTCGGCACACAGAC | 108 |
|  | Reverse | AGGTTTTACTGTGAGGGCGG |  |
| *SEZ6* | Forward | GAGTTGGAGGTGTGTGCGTA | 138 |
|  | Reverse | TGTGGGTCTCCTCGTCTCAT |  |
| *NCAM* | Forward | GGTGGTGGCTTGTTTGTTGG | 117 |
|  | Reverse | TGTGTATGTGTGAGGGGTGC |  |
| *RIMS2* | Forward | GCACAACGCTAAACGAGGAG | 123 |
|  | Reverse | CGGTGCCATCTTTCATCCGT |  |
| *FGF13* | Forward | AGGGAGCGAGAGAAGTCCAA | 118 |
|  | Reverse | TGGAGCCGAAGAGTTTGACC |  |
| *CACNB3* | Forward | CACGAGCAGACCTTCGGATT | 162 |
|  | Reverse | TGTGTGGGAGTGACGACAAG |  |
|  |  |  |  |
